# Coronavirus-19 Multisystem Inflammatory Syndrome in Children (MIS-C): A Pediatric Simulation Case for Residents, Fellows, and Advanced Practice Providers

**DOI:** 10.15766/mep_2374-8265.11180

**Published:** 2021-08-16

**Authors:** Dhritiman Gurkha, Katie Cashen, Paul Patek, Karima Lelak, Kelly Levasseur

**Affiliations:** 1 Fellow, Department of Pediatric Emergency Medicine, Oakland University William Beaumont School of Medicine; 2 Associate Professor, Department of Pediatrics, Central Michigan University College of Medicine; 3 Fellow, Department of Pediatrics, Central Michigan University College of Medicine

**Keywords:** Pediatrics, Pediatric Emergency Medicine, COVID-19, Multisystem Inflammatory Syndrome in Children, Shock, Case-Based Learning, Clinical/Procedural Skills Training, Simulation

## Abstract

**Introduction:**

A rare but serious condition often requiring intensive care, multisystem inflammatory syndrome in children (MIS-C) is characterized by hyperinflammatory shock related to the SARS-CoV-2 pandemic. This resource teaches residents, pediatric emergency medicine fellows, and advanced practice providers who care for children to recognize and manage MIS-C and associated sequelae while applying the basic principles of pediatric resuscitation.

**Methods:**

The simulation case was based on a real patient who presented to the emergency department with fever, rash, and cardiogenic shock. We designed the scenario to be used with a high-fidelity school-age mannequin in an emergency center resuscitation room or simulation lab. The case took 25 minutes to run, followed by a 15- to 20-minute debrief session. Personnel required for the case included a simulation technician, case instructor, emergency department nurse, parent, and consultant. Learners had to recognize the syndrome and treat the resultant shock and arrhythmia with a combination of vasopressors, antiarrhythmics, and defibrillation. Afterward, learners participated in a formal debriefing session and completed a written evaluation.

**Results:**

Twenty-five learners (six pediatric emergency medicine fellows, 12 residents, and seven advanced practice providers) participated in the scenario over a 3-month period. The written evaluation was completed by 20 of the 25 participants; all 20 felt their confidence, comfort, and knowledge regarding the topic had increased, with an average score of 5 (*strongly agree*) on a 5-point Likert scale.

**Discussion:**

This simulation case offers an effective experience for learners to become comfortable and confident in recognizing and managing MIS-C.

## Educational Objectives

By the end of this activity, learners will be able to:

1. Develop an organized approach to the evaluation of a child with multisystem inflammatory syndrome in children (MIS-C).
2. Demonstrate an approach to effectively manage MIS-C.
3. Identify and diagnose cardiogenic shock in a child.
4. Demonstrate the ability to perform a pediatric resuscitation based on Pediatric Advanced Life Support algorithms.

## Introduction

Severe acute respiratory syndrome coronavirus 2 (SARS-CoV-2) causing coronavirus disease 2019 (COVID-19) was initially described as a mild or asymptomatic disease in children with rare associated mortalities; we now know children with COVID-19 can become very ill and require intensive care.^1–3^ Recently, multiple pediatric centers reported an increase in patients presenting with hyperinflammatory shock and multisystem involvement with features similar to Kawasaki disease, now recognized as multisystem inflammatory syndrome in children (MIS-C) related to the SARS-CoV-2 pandemic.^4–6^ Most children with MIS-C presented to emergency departments with severe symptoms days to weeks after becoming infected with COVID-19. Many of the patients required intensive care; fortunately, short-term clinical recovery was nearly universal with prompt recognition and management.^7,8^

A diagnosis of MIS-C is made based on case definitions put forth by the Centers for Disease Control and Prevention (CDC) and the World Health Organization. The diagnosis is defined as a patient meeting all of the following clinical criteria: (1) age less than 21 years, (2) fever greater than 38 °C or a report of subjective fever lasting 24 hours or more, (3) laboratory evidence of inflammation, (4) severe illness requiring hospitalization, (5) two or more organ systems involved, (6) no alternative plausible diagnosis, and (7) recent or current SARS-CoV-2 infection or exposure.^9,10^ Laboratory evidence of inflammation is defined as an elevated C-reactive protein, erythrocyte sedimentation rate, fibrinogen, procalcitonin, D-dimer, ferritin, lactic acid dehydrogenase, or interleukin-6 level.^9,10^ SARS-CoV-2 infection or exposure is defined as any of the following: (1) positive SARS-CoV-2 polymerase chain reaction, (2) positive serology for SARS-CoV-2, (3) positive antigen test, or (4) COVID-19 exposure within the 4 weeks prior to the onset of symptoms.^9,10^ In conjunction with the above case definition, it is important to understand the clinical features of typical and atypical Kawasaki disease as these features fall within the spectrum of MIS-C. A substantial proportion of patients diagnosed with MIS-C meet diagnostic criteria for Kawasaki disease, which are (1) fever lasting at least 5 days, (2) bilateral conjunctival congestion, (3) changes of the lips and oral cavity, (4) polymorphous exanthema, (5) changes of peripheral extremities, and (6) acute nonpurulent cervical lymphadenopathy.^11^

The targeted treatment of MIS-C includes intravenous immunoglobulin (IVIG), systemic steroids, biologics (anakinra and tocilizumab), and supportive care. In the acute care setting, the provider must recognize the shock-like state associated with the illness and provide resuscitation as outlined by the Surviving Sepsis Campaign and Pediatric Advanced Life Support (PALS) algorithms.^12^

We designed this case for residents and fellows who care for children with the goal of providing an educational experience that addresses a knowledge gap in a novel disease process. In order to appropriately treat patients with MIS-C, rapid recognition is imperative. Also important is effective communication with subspecialists, including infectious disease, cardiology, and intensive care physicians, and implementation of foundational pediatric emergency medicine principles. As this is a new and rare disease process, few learners have been exposed to patients with MIS-C during their training, which could lead to delayed recognition and poor outcomes. Simulation has been proven to be an effective method to introduce learners to uncommon yet high-risk scenarios while providing a safe environment for physicians and trainees to to practice and reflect on their management, making simulation an excellent avenue for preparing learners to manage the challenges associated with MIS-C.^13,14^ The educational technique used in this simulation case is grounded in cognitive learning theory.^15^ This theory helps learners to understand why they did what they did during a simulation case and to learn and retain the knowledge better than in a classroom setting or in a simulation case without a debrief session. In this technique, learners participating in a simulation case are allowed to make errors in order to learn from them. Also, questioning learners about why they managed the simulated patient a certain way allows for reflection and learning in a safe environment.^16^

We specifically designed the scenario for health care practitioners working with critically ill patients, while emphasizing quick, active thinking and procedural skills. In a search of *MedEdPORTAL,* we found no published resources concerning shock in a pandemic situation; specifically, there were no published resources pertaining to MIS-C. One resource we found focused on Kawasaki disease, and others focused on hypovolemic and septic shock.^17–19^ These publications were designed to stress the importance of physical exam, rapid recognition, and consultation. We present a challenging case incorporating the basic principles of shock management in pediatric patients with a novel disease that can be managed effectively to complete clinical recovery with careful intensive care but has the potential to be rapidly fatal if not identified or managed promptly and effectively.^7,8,20^

## Methods

### Development

MIS-C due to COVID-19 is an important diagnosis to consider in critically ill children. This simulation case taught learners to recognize MIS-C in the setting of fever, rash, and cardiogenic shock. The diagnosis was made by combination of history, physical exam, and lab results, based on the CDC's MIS-C case definition. We based the case on an actual patient seen in the emergency department so that our learners would be exposed to a realistic MIS-C scenario. The case was developed by pediatric emergency medicine physicians in collaboration with pediatric intensivists. The simulation session took place in residents’ and fellows’ pediatric emergency medicine rotation during a scheduled weekly conference time. The family practice residents did this rotation during their third year of training, whereas the emergency medicine residents and pediatric residents rotated during their second and third years. The pediatric emergency medicine fellows all participated in this case regardless of their year of training. The advanced practice providers (APPs) had quarterly simulation sessions, and this case was included in one of them. The background knowledge expected of all participants was to be Basic Life Support (BLS) and PALS certified and aware of how to diagnose MIS-C in the pediatric population. The lead instructor had to have a solid understanding of how to recognize and treat ill children, ideally being an emergency medicine physician, pediatric emergency medicine physician, or other acute care provider with these skills.

### Equipment/Environment

The following equipment was available and utilized to optimize the case presentation:

• High-fidelity mannequin (school-age child sized; a Laerdal SimJunior was used at our institution), placed in a gown and covered with a blanket. This mannequin was programmable to reflect certain physical exam findings, for instance, auscultatory findings such as wheezes or crackles, pulse variability based on blood pressure, and so on. The mannequin was placed on a gurney. Clothing was left on the mannequin to make discovery of the rash more difficult for advanced learners.
• Intubation supplies: bag valve mask, suction, Macintosh and/or Miller laryngoscope blade (sizes 1 and 2), multiple pediatric cuffed endotracheal tubes (sizes 4.5, 5.0, and 5.5), Glidescope or other advanced airway devices if available.
• Adolescent non-rebreather mask and nasal cannula.
• Peripheral intravenous lines and tubing.
• Intravenous normal saline (500-cc bag).
• Medication vials: induction medications (midazolam, fentanyl, ketamine, or etomidate), paralytics (succinylcholine, vecuronium, or rocuronium).
• Color-coded length-based tape measure (Broselow tape was used at our institution).
• Bedside monitor: heart rate, respiratory rate, cardiac monitor, blood pressure, temperature.
• Case templates needed to run the case:
  ○ The simulation case template (Appendix A).
  ○ Imaging studies (Appendix B): a chest radiograph depicting enlarged cardiomediastinal silhouette and prominent vascular markings. Radiographs were provided to learners if they specifically asked for them.
  ○ Laboratory results (Appendix C) were provided to learners upon their request. It was important to have each set of laboratory values on a different sheet (e.g., complete blood count on one sheet and electrolytes on another) so that learners could be provided only with what they specifically requested. Please note that laboratory tests not listed in this handout were normal. Note: Both imaging studies and laboratory studies can be laminated for repeat use.

### Personnel

Personnel involved in the simulation included the following:

• Parent/caregiver: This role was played by the case instructor and functioned to answer participant questions regarding the patient's history as well as to add additional distraction. Parental inquiry often helped instructors understand why learners were performing certain actions in real time. For advanced trainees, parents were frequently questioned to emulate real-life situations.
• Emergency department nurse (ED RN): This role was played by a pediatric ED RN but could also be played by a case instructor, senior pediatric emergency medicine fellow, or any other senior-level trainee or physician. The nurse functioned in a typical nursing role and carried out actions per the learners’ request. The nurse could also distract advanced learners by getting overwhelmed or being unable to successfully perform tasks requested, for example, securing an intravenous line.
• Consultants: The learners could ask for the aid of a consultant from any subspecialty. At our institution, participants placed a consult by using a phone or verbalized whom they wanted to consult. We used a working phone that called an instructor, who provided a scripted response in real time. In some cases, the consultant was unavailable in an effort to force the learners to make necessary decisions or perform necessary procedures.
• Case instructors: Two additional instructors were needed, one to manage the mannequin's response to treatment and the other to answer questions regarding the physical exam and act as consultant when called. This second instructor also took notes on the learners’ progression through the case for the debriefing session.

### Implementation

The lead instructor read over the documents and appendices needed to run this case, which took about 20 minutes. The simulation was performed in one of our trauma bay simulation rooms. The simulation room was set up with the mannequin on a bed with the proper equipment ready for the simulation case. The lead instructor ensured that the learners were all familiar with the room and equipment. Learners were educated on how the monitor and high-fidelity mannequin worked. This preparation took about 15 minutes. Teams of four to five learners participated in the simulation. They were told that the triage sheet with vitals and patient's name (Appendix D) would be placed on the end of the bed. The team was then told that a patient was in one of the critical beds in the emergency department and that they had 2 minutes to assign roles and evaluate the patient. The lead instructor went into the control room, which had a one-way mirror into the simulation room. The lead instructor changed the respiratory and cardiovascular findings of the mannequin and other vital signs as necessary and could talk to the team over a loudspeaker, if needed. The simulation lasted about 20 minutes from start to finish. The group then moved to a conference room to discuss the case, including the debriefing questions (Appendix E). The critical action checklist (Appendix F) was completed. At the end of the simulation case and debrief session, all learners filled out a learner evaluation of the simulation case (Appendix G).

### Assessment

During the simulation, the case instructors assessed the participants with formative assessment (using Appendix F), and feedback was provided verbally. Instructors noted times that specific actions were completed to inform the learners during the debrief session. They also focused on the learners’ ability to form a team and communicate successfully. Instructors made notes of roles being assigned by the team leader at the start of the case. Team interaction was evaluated based on whether closed-loop communication was occurring throughout the case. In addition, team leaders were evaluated on their summarizing ability to keep team members in the loop while reviewing the case progression in real time. The critical action checklist (Appendix F) was completed by the case instructors following the conclusion of the debrief session. Learners were primarily assessed on whether they completed the critical actions. These critical actions were chosen by the instructors to mimic a real resuscitation for a child in cardiogenic shock, the components of which are time sensitive and impact patient outcomes. The debrief session (see below) was framed around formative feedback provided to the learners as a group based on their individual performances. The evaluations from the learners were used to improve future iterations as well as to obtain learners’ assessment of whether they had achieved the objectives.

### Debriefing

Upon case completion, the learners and instructors moved to an area where they could comfortably discuss and evaluate the case's progression, which typically took about 20 minutes. The facilitator first asked the group to summarize the case. Initial discussion focused on eliciting learner opinion in regard to case discovery and overall management. The group was then asked to discuss what the learners felt they had done well and what they would do differently. The facilitator helped the group recognize the key learning elements of the case if the group did not recognize them all (Appendix E contained a list of debrief questions to ask learners). Debriefing questions covered the most important learning elements and gave the facilitators a framework to ensure they captured the important educational objectives one at a time. Additional debriefing questions followed to prompt learners to further discuss any additional educational objectives that had not been explored.

We used the critical action checklist (Appendix F) to identify whether learners were able to achieve the educational objectives. The groups were scored in one of three categories: complete, partial, or none. If they completed all actions required, they achieved complete status; if they completed only some of the actions, they achieved partial status. The critical action checklist was created by instructors to mimic components of a real resuscitation for a child with cardiogenic shock, so meeting these actions suggested that the resource was effective. The critical action checklist was completed following the debrief session by the lead instructor, as well as by other instructors present for the case. This included peer evaluation completed by senior fellows or residents as the Accreditation Council for Graduate Medical Education encourages co-resident evaluation.

## Results

Between May 2020 and July 2020, 25 learners participated in the simulation case. These included 12 residents in emergency medicine, family practice, and pediatrics; six pediatric emergency medicine fellows; and seven APPs. The pediatric emergency medicine fellow, senior emergency medicine resident, or APP was the leader of the case. The other learners played other roles in the case, such as airway, talking to the family member, or compressions.

During our study period, six simulation cases were conducted with distinct groups of learners (four to six learners in each group). On the critical action checklist, all groups achieved either complete or partial status for each action. The time to critical action varied among groups, with more-senior learners initiating treatment earlier.

Our objectives included developing an organized approach to the evaluation of a child with MIS-C, identifying and diagnosing a patient in cardiogenic shock, and demonstrating an approach to managing MIS-C. The critical action checklist specifically identified each of these objectives. Four of the six teams attained complete status on the critical actions of recognizing MIS-C and potential for cardiogenic shock and demonstrating appropriate management of MIS-C. The other two teams attained partial status, missing the need for IVIG and possible thromboprophylaxis, as well as demonstrating delayed recognition of cardiogenic shock. All teams delayed in donning personal protective equipment (PPE), and so, all received partial status on the critical action checklist.

We also solicited direct feedback from participants via Appendix G, the learner evaluation of the simulation case. Items included the following: “Simulation case was appropriate for level of training,” “Scenarios were representative of cases we could see,” “Debriefing was helpful for understanding the case,” “Participating in this simulation case has increased my confidence, comfort level, and knowledge,” and “Participating in this simulation case has helped facilitate team building.” Self-report via a 5-point Likert scale (1 = *strongly disagree,* 5 = *strongly agree*) demonstrated that learners strongly agreed with all of these statements. All items except for the appropriateness for level of training received a 5.0. Five learners felt the case was too difficult for them, and so, the score for that statement was 4.7 (see the Table). The only feedback that we received from an open-ended question was that the case was too difficult for some APPs, pediatric residents, and family practice residents. Respondents felt that they could recognize a patient with MIS-C; however, managing a patient with shock and intubation was beyond their training.

**Table.**
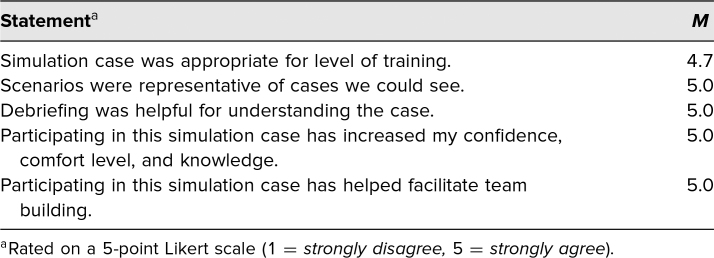
Mean Scores for Content and Delivery

## Discussion

The COVID-19 pandemic has created an unfamiliar territory for health care providers. With each patient case encountered, the clinical dimensions of this disease process are becoming evident. Unfortunately, thanks to the current climate, many traditional educational opportunities have been placed on hold. Despite residents and fellows continuing to care for these patients, opportunities to learn outside of clinical care are limited. This simulation scenario was created in order to extend this new knowledge base and to provide simulation-based learning about COVID-19 and MIS-C for physicians and APPs caring for children during an evolving pandemic.

The simulation was developed with a range of clinical competencies to accommodate learners from different levels of training and was implemented in the setting of an existing simulation curriculum. This scenario fostered practice in communication and leadership skills, in addition to improving proficiency and confidence regarding pediatric resuscitation and critical procedures. The final diagnosis for our case was a new disease entity, but the sequence of resuscitation in a child with cardiogenic shock was standard and built on existing knowledge acquired in medical school and during training. Our identification of critical actions was based on PALS algorithms and American Heart Association recommendations for resuscitation of children with congenital and acquired heart disease. Our learners were able to use and build on the fundamentals of pediatric resuscitation, including PALS, to care for a critically ill child.

While all the learners indicated that they were familiar with MIS-C, only half of the teams recognized that this patient had MIS-C early in the case, and delayed recognition was apparent. The delay in time to recognition prevented teams from wearing appropriate PPE as well as from anticipating the rapid deterioration of the patient. Additionally, we observed that a majority of teams focused their efforts on trying to identify treatment modalities for MIS-C, rather than on crucial resuscitation of a child with cardiogenic shock. In the standard resuscitation sequence, BLS and PALS critical actions must occur prior to treatments for MIS-C in order to stabilize the patient. All learners stated that this case was valuable because they had not yet cared for a patient with MIS-C. Additionally, debriefing highlighted that learners felt overwhelmed by their lack of experience in assessing a patient with MIS-C. The presentation of children with COVID-19 is dynamic, and it is imperative for learners to understand that this is an evolving disease process and the presentation may be variable. Evaluating the simulation, our learners gave the highest scores to their perception of improved confidence and knowledge acquisition.

This scenario is limited by many of the factors that make simulation challenging. Conjunctivitis and a realistic rash can be difficult to represent on a simulation mannequin; however, this difficulty can be overcome with moulage. In addition, the generalizability of this simulation scenario is fluid due to the constantly evolving information we have related to COVID-19. Our understanding and the most common clinical presentation of MIS-C will likely be modified as we gain more knowledge of this disease in different patient populations and countries. Additionally, we did not use a validated evaluation tool for the simulation. We relied on learner self-report via Likert scale to assess perceived benefit of the simulation. We feel that the learners gave the simulation case very high marks because it was implemented early on during the COVID pandemic and they were happy to be learning about MIS-C in a simulation lab. It was (and continues to be) a highly stressful time for them, and the case allowed them to feel more comfortable and confident when caring for pediatric patients with this novel disease.

Future directions for this work will be guided by the medical literature surrounding MIS-C. As our knowledge of this disease process increases, the simulation may be modified to focus on more objective task-based evaluation and continuing care of the patient, with transition of care to the ICU and cannulation to venoarterial extracorporeal membrane oxygenation with full PPE. We plan to repeat the scenarios with the new wave of COVID-19 to assess retention of knowledge and meeting of the case objectives, as well as to implement a validated evaluation tool for donning PPE and clinical performance.

## Appendices


Simulation Case.docxImaging Studies.docxLaboratory Studies.docxTriage Sheet.docxDebriefing Questions.docxCritical Action Checklist.docxLearner Evaluation of Mock Code.docx
*All appendices are peer reviewed as integral parts of the Original Publication.*
