# Supplementary material for: Coronavirus-19 Multisystem Inflammatory Syndrome in Children (MIS-C): A Pediatric Simulation Case for Residents, Fellows, and Advanced Practice Providers
Source: MedEdPORTAL. 2021 Aug 16;17:11180. doi: 10.15766/mep_2374-8265.11180 (PMC8364930; doi:10.15766/mep_2374-8265.11180)

**Labs CBC/ CMP:**

| Laboratory test | Units / (Normal range) | Admission |
| --- | --- | --- |
| Hemoglobin | g/dL (11.6-15.1) | 10.9 |
| White blood cells | mm^3^ (4.1-11.3) | 13.3 |
| Neutrophil | % | 75 |
| Bands | mm^3^ (0-0.5) | 1.2 |
| Lymphocyte | % | 15 |
| Platelets | mm^3^ (130-450) | 225 |
| Sodium | mm/L (136-145) | 118 |
| Creatinine | mgm/dL (0.3-0.6) | 1.09 |
| Alanine Aminotransferase | U/ L ( 7-52) | 25 |
| Albumin | g/dL (3.8-4.7) | 2.8 |
| Lactate dehydrogenase | U/ L (140-271) | 794 |

**Labs – COVID**

| Laboratory test | Units / Normal range | Admission |
| --- | --- | --- |
| SARS-CoV 2 IgG Antibodies |  | Positive |
| Laboratory test | Units / Normal range | Admission |
| D- Dimer | mg/L (<0.5) | 4.21 |
| Ferritin | ng/mL ( 11-306.8) | 699.5 |
| High Sensitivity Troponin | ng/L (3-17) | 114 |
| Fibrinogen | mg/dL (186-466) | 834 |
| C –Reactive Protein | mg/dL (<5) | 450 |


Supplement: Supplementary file 1 — Simulation Case.docxImaging Studies.docxLaboratory Studies.docxTriage Sheet.docxDebriefing Questions.docxCritical Action Checklist.docxLearner Evaluation of Mock Code.docx [file mep_2374-8265.11180-s001.zip › C. Laboratory Studies.docx]
