# Supplementary material for: Coronavirus-19 Multisystem Inflammatory Syndrome in Children (MIS-C): A Pediatric Simulation Case for Residents, Fellows, and Advanced Practice Providers
Source: MedEdPORTAL. 2021 Aug 16;17:11180. doi: 10.15766/mep_2374-8265.11180 (PMC8364930; doi:10.15766/mep_2374-8265.11180)
Supplement: Supplementary file 1 — Simulation Case.docxImaging Studies.docxLaboratory Studies.docxTriage Sheet.docxDebriefing Questions.docxCritical Action Checklist.docxLearner Evaluation of Mock Code.docx [file mep_2374-8265.11180-s001.zip › D. Triage Sheet.docx]

**Pediatric Emergency Department - Room 408**

Patient Name: Shelia Smith

MRN: 8945562004

Age: 6 y/o

Parent: Heather Smith

Chief complaint: Fever, rash

**Triage Vitals:**

HR 145

RR 35

98% on RA

BP 100/60

T 39°C
