# Supplementary material for: Coronavirus-19 Multisystem Inflammatory Syndrome in Children (MIS-C): A Pediatric Simulation Case for Residents, Fellows, and Advanced Practice Providers
Source: MedEdPORTAL. 2021 Aug 16;17:11180. doi: 10.15766/mep_2374-8265.11180 (PMC8364930; doi:10.15766/mep_2374-8265.11180)
Supplement: Supplementary file 1 — Simulation Case.docxImaging Studies.docxLaboratory Studies.docxTriage Sheet.docxDebriefing Questions.docxCritical Action Checklist.docxLearner Evaluation of Mock Code.docx [file mep_2374-8265.11180-s001.zip › E. Debriefing Questions.docx]

**Debriefing Questions**

The facilitator will ask the participants to critique their management of the patient. Potential questions or points for discussion during debriefing are:

| **Key Question** | **Points to Discuss** |
| --- | --- |
| 1. How well did you form a team? | Team leader needs to specify roles for each team member. Leaders must have closed loop communication with their team. |
| 2. Did you recognize the H&P and PE as concerns for Covid infection and possibly MIS-C? | Patient had: Fever for >5d, conjunctival injection, cervical lymphadenopathy, edema of both upper and lower extremities, maculopapular rash alongside positive SARS-CoV-2 contact. |
| 3. Did you recognize that the patient was having difficulty breathing? How quickly did you implement an intervention and was it appropriate? | Patient (may have) received a fluid bolus for tachycardia and began having worsening tachypnea requiring oxygen therapy and eventually intubation. Airway management is a cornerstone of resuscitation and given the rapid deterioration of the patient maintaining their airway and supporting ventilation is a priority. The patient should be intubated even if there is a risk for cardiac collapse during the intubation because once the patient is intubated shock should improve because about 40% of cardiac output goes toward work of breathing. |
| 4. What is your interpretation of vital signs/EKG/Echo/change in clinical course leading to diagnosis of cardiogenic shock? | Guide the learners to recognize initial tachycardia to obtain the CXR and EKG to evaluate for cardiomegaly and myopericarditis, respectively. Consider point of care ultrasound (POCUS) echocardiogram to evaluation for cardiac contractility and pericardial effusion. Learners need to be alerted about the hypotension and deterioration after more than one 20cc/kg fluid bolus, a clinical feature of cardiogenic shock. |
| 5. What was your interpretation of the change in vital signs/EKG with transition of heart rhythm to VT? | Using the AHA PALS card and EKG, discuss algorithm-based management for VT with a pulse. Specify appropriate use of synchronized cardioversion, pharmacological management. Highlight the importance of timely pulse checks. If pulseless VT, delineate steps of defibrillation on specific bedside device |
| 6. What was your final disposition for the patient? | The patient requires close cardiorespiratory monitoring which can only be performed at a PICU and with ECMO capabilities, if needed. If ECMO capabilities are not present at your facility it is imperative that the patient is transferred by ground or by flight with a highly trained pediatric transport to a higher level of care for definitive management. |
