# Supplementary material for: Coronavirus-19 Multisystem Inflammatory Syndrome in Children (MIS-C): A Pediatric Simulation Case for Residents, Fellows, and Advanced Practice Providers
Source: MedEdPORTAL. 2021 Aug 16;17:11180. doi: 10.15766/mep_2374-8265.11180 (PMC8364930; doi:10.15766/mep_2374-8265.11180)
Supplement: Supplementary file 1 — Simulation Case.docxImaging Studies.docxLaboratory Studies.docxTriage Sheet.docxDebriefing Questions.docxCritical Action Checklist.docxLearner Evaluation of Mock Code.docx [file mep_2374-8265.11180-s001.zip › F. Critical Action Checklist.docx]

| **Simulation Title**  **Cardiogenic Shock due to Multisystem Inflammatory Syndrome in Children (MIS-C) associated with Coronavirus Disease (COVID-19)** | | | | |
| --- | --- | --- | --- | --- |
| **Participant Name(s):** | | | | |
| **Date:** | | | | |
| **Key Simulation Events**  **(list of learning objectives and/or key steps toward achievement)** | **Complete** | **Partial** | **Incomplete** | **Comments** |
| 1. Formation of a team: Team leader established, team member role identified by closed loop communication |  |  |  |  |
| 2. Recognized MIS-C and potential for cardiogenic shock: CDC case definition for MIS-C and identified hypotension in response to fluid boluses |  |  |  |  |
| 3. Appropriate management of airway: Nonrebreather mask with supplemental oxygen and escalate appropriately to secure advanced airway while using BVM; appropriate RSI agents |  |  |  |  |
| 4. Appropriate management of MIS-C: Consider IVIG +/- Steroids/Biologics, Started PO Aspirin, verbalize need for thromboprophylaxis |  |  |  |  |
| 5. Created a disposition for the patient:  Admit or transfer to PICU, verbalize possible need for ECMO |  |  |  |  |
| 6. Correct teams consulted: Cardiology, Pediatric Intensive Care, +/- Neurology, +/- Hematology |  |  |  |  |
| 7. Wear appropriate Personal Protective Equipment (N95 or equivalent respirator, gloves, gown, caps): This must be worn by all team members |  |  |  |  |
| 8.Did the care improve the patient’s condition? Placed the patient on cardiorespiratory monitor. Secured airway with appropriate RSI drugs. Initiated PALS based management for Ventricular Tachycardia. |  |  |  |  |
