# Supplementary material for: Coronavirus-19 Multisystem Inflammatory Syndrome in Children (MIS-C): A Pediatric Simulation Case for Residents, Fellows, and Advanced Practice Providers
Source: MedEdPORTAL. 2021 Aug 16;17:11180. doi: 10.15766/mep_2374-8265.11180 (PMC8364930; doi:10.15766/mep_2374-8265.11180)
Supplement: Supplementary file 1 — Simulation Case.docxImaging Studies.docxLaboratory Studies.docxTriage Sheet.docxDebriefing Questions.docxCritical Action Checklist.docxLearner Evaluation of Mock Code.docx [file mep_2374-8265.11180-s001.zip › G. Learner Evaluation of Mock Code.docx]

**Mock Code evaluation form**

1 strongly disagree, 3 neutral and 5 strongly agree

1. Mock code was appropriate for level of training 1 2 3 4 5
2. Adequate staff to run the mock code 1 2 3 4 5
3. Scenarios were representative of cases we could see 1 2 3 4 5
4. Debriefing was helpful for understanding the case 1 2 3 4 5
5. Participating in this mock code has increased my confidence, comfort level and knowledge 1 2 3 4 5
6. Participating in this mock code has helped facilitate team building

1 2 3 4 5

Suggestions for case improvement:____________________________________________________________________________________________________________________________________________________________________________
