# Supplementary material for: Coronavirus-19 Multisystem Inflammatory Syndrome in Children (MIS-C): A Pediatric Simulation Case for Residents, Fellows, and Advanced Practice Providers
Source: MedEdPORTAL. 2021 Aug 16;17:11180. doi: 10.15766/mep_2374-8265.11180 (PMC8364930; doi:10.15766/mep_2374-8265.11180)
Supplement: Supplementary file 1 — Simulation Case.docxImaging Studies.docxLaboratory Studies.docxTriage Sheet.docxDebriefing Questions.docxCritical Action Checklist.docxLearner Evaluation of Mock Code.docx [file mep_2374-8265.11180-s001.zip › A. Simulation Case.docx]

| Appendix A: MedEdPORTAL Simulation Case Template    SIMULATION CASE TITLE: Cardiogenic Shock due to Multisystem Inflammatory Syndrome in Children (MIS-C) associated with Coronavirus Disease (COVID-19)    AUTHORS: Dhritiman Gurkha, Katie Cashen, Paul Patek, Karima Lelak, Kelly Levasseur    LEARNER AUDIENCE: Pediatric Emergency Medicine (PEM) fellows, Residents (Emergency Medicine, Pediatrics, Family Medicine, Internal Medicine/Pediatrics) | | | | |
| --- | --- | --- | --- | --- |
| PATIENT NAME: Shelia Smith    PATIENT AGE: 6 years old    CHIEF COMPLAINT: Fever and Rash    PHYSICAL SETTING: Emergency Department (ED) | | | | |
|  | | | | |
| Brief narrative description of case | 6 yo female with fever and rash. Learner should have Pediatric Advanced Life Support (PALS) skills and have basic knowledge about Cardiogenic shock, Extracorporeal Membrane Oxygenation (ECMO), SARS-CoV-2 and the complications including multisystem inflammatory syndrome in children (MIS-C) | | | |
| Primary Learning Objectives | 1. Develop an organized approach to the evaluation of a child with multisystemic inflammatory syndrome in children (MIS-C)  2. Demonstrate an approach to effectively manage MIS-C  3. Identify and diagnose cardiogenic shock in a child  4. Demonstrate the ability to perform a pediatric resuscitation based on PALS algorithms | | | |
| Critical Actions | 1. Evaluate patient and determine to have features concerning for COVID-19. 2. Place patient on cardiorespiratory monitor and recognize tachycardia 3. IV ordered along with labs, Electrocardiogram, Echocardiogram and Chest radiograph (CXR) 4. Recognize signs of shock - hypotension and worsening tachycardia after IV fluid bolus given for tachycardia 5. Recognize mild cardiomegaly on CXR 6. Identify respiratory distress and deteriorating mental status as cardiogenic shock after developing hypotension in response to fluid bolus 7. Intubate for airway protection and initiate Epinephrine Drip for hypotension 8. Identify runs of Ventricular Tachycardia and initiate IV Amiodarone drip 9. Transfer patient to Pediatric Intensive Care Unit (PICU)/ECMO center | | | |
| Learner Preparation or Prework | Learners should have the knowledge of PALS and  Learners should be aware of the following:  Cardiogenic Shock  COVID-19 and MIS-C    No other pre reading or training needed prior to participating in this case. | | | |
| Initial Presentation | | | | |
| Initial vital signs | HR 145, RR 35, SpO2 100% on RA, BP 100/60, T 39°C | | | |
| Overall Setting and Appearance | Learners enter a room that looks like a critical care bed in an emergency department. They will find the mannequin in a supine position with the patient's mother and nurse at bedside. They will be told that their patient seems to be in distress and has conjunctival injection and a diffuse Maculopapular Rash | | | |
| Confederates (e.g., standardized participants) and their roles in the room at case start | Patient’s Mother (PM) and Bedside Nurse (RN) present at bedside. PM and RN roles can be played by two different faculty, simulationists or trainees.  PM: My daughter does not feel well. She has been having a fever for the last 6 days.  RN: Let me get the physicians | | | |
| HPI | 6 y/o girl, previously healthy, presents from home with chief complaint of “Fever” (Printed out on piece of paper kept on the bed)  (Upon asking mother, what brings your daughter to the ED?)  PM: Sheila has been having a fever for the last 6 days in the range of 38-40C. I don’t have any medicine in the house and didn’t want to go to the drug store to get Tylenol because of the corona outbreak.  (Upon asking any other complaints with the fever)  PM: Her eyes are starting to look kind of red and her hands and feet are puffy. And today she’s complaining of abdominal pain and not eating as much so they decided to come to the ER. I also found out that someone at her daycare tested positive for Coronavirus yesterday. (For senior PEM Fellows: PM – My husband has been coughing for the last 1 week, do not given information about possible covid contact) | | | |
| Past Medical/Surgical History | Medications | Allergies | Family History | |
| None | Acetaminophen  Ibuprofen | None | None | |
| Physical Examination | | | | |
| General | Non-toxic but appears tired, interactive | | | |
| HEENT | Normocephalic/Atraumatic, Tympanic Membranes clear bilaterally, conjunctival injection | | | |
| Neck | Shotty cervical lymphadenopathy | | | |
| Lungs | Tachypnea but clear to auscultation bilaterally, no respiratory distress or retraction | | | |
| Cardiovascular | Tachycardia, S3 gallop, Regular rhythm | | | |
| Abdomen | Soft, non-distended, no rigidity, no guarding | | | |
| Neurological | Alert and Oriented x3, Cranial Nerves 2-12 grossly intact | | | |
| Skin | Diffuse Maculo-papular rash, no excoriations or drainage. Non-pitting edema of bilateral upper and lower extremities, no erythema, no bruising. | | | |
| GU | Normal for age | | | |
| Psychiatric | Appropriate affect for age | | | |
| Instructor Notes - Changes and CASE Branch Points | | | | |
| Intervention / Time point | | Change in Case | | Additional Information |
| Start/ Timeline | Expected Performance  (Learning Objectives) | Achievement of Objective  (Trigger to move to next state) | | Learning Cues |
| Simulation Start  (Minutes 0 – 5) | Patient is evaluated and determined to have features concerning for MIS-C ( Learners expected to verbalize the CDC MIS-C criteria)    IV ordered along with labs, EKG, Echo and CXR, covid testing | IV ordered and pt is placed on cardiorespiratory monitor, noted to be tachycardic with HR in 140’s    Aspirin and IVIG may be considered (+/- in conjunction w/ consult request) | | Nurse may ask if they would like any lab work/imaging/EKG/meds started    If appropriate PPE is not donned,  the nurse must ask the team about PPE and show them where it is  kept. |
| Critical Change 1    5-15 minutes | If pt is given a fluid bolus > 10mL/kg for the tachycardia; tachycardia worsens, pt becomes hypotensive. If they give 5mL/kg pt may initially improve and then get worse.    Xray is interpreted as mild cardiomegaly    COVID-19 +, mild leukocytosis, BNP mildly elevated, Troponin mildly elevated, otherwise unremarkable. At this point, learners need to verbalize MIS-C Confirmed | Pt is diagnosed with MIS-C, c/b evolving cardiogenic shock, Epi drip initiated starting at 0.05 mcg/kg/min    Patient starts to have more difficulty breathing and becomes less awake. Patient is intubated and also started on Epinephrine drip for cardiogenic shock. The appropriately sized equipment/meds needs to be verbalized based on Length based tape for the mannequin/patient | | Nurse may need to report pt is now less interactive, change in vital signs    Nurse may need to prompt for RSI/post-sedation med |
| Critical Change 2    15-20 minutes | Patient is re-evaluated and determined to be having runs of Ventricular Tachycardia based on the monitor  (HR 200s)  Learners expected to verbalize wide QRS with tachycardia and verify presence of pulse | Amiodarone drip is started w/ improvement (bolus followed by infusion)    PICU or Transfer center for ECMO or cardiac transplant | | Nurses asks if the pt can be transferred once the patient is:  · Intubated  · On Epi drip (or similar)  Amio drip (or similar) |
|  |  |  |  |  |

Ideal Scenario Flow:

Learners enter the room and identify that the patient is tired appearing and tachypneic. They immediately place the patient on a bedside monitor and recognize the patient is febrile and tachycardic. They place the patient on supplemental oxygen, obtain intravenous access and point of care Glucose. Upon obtaining focused history and physical exam, they consider Kawasaki’s Disease and COVID-19 possibly MIS-C. They obtain appropriate labs, EKG, imaging while giving a rapid IV fluid bolus of 20ml/kg for tachycardia. They re-evaluate the patient and recognize that tachycardia has worsened and the patient is now hypotensive with deterioration in mental status. They diagnose cardiogenic shock and promptly initiate IV epinephrine drip while securing the airway with advanced airway using appropriate rapid sequence induction drugs. After confirming endotracheal tube placement, the learners keep the patient sedated and paralyzed. Following this the patient starts to have runs of ventricular tachycardia (VT) which is promptly recognized and managed using the PALS algorithm. Once the Amiodarone drip is initiated, the patient is transferred to the PICU/ECMO center for further management.

Anticipated Management Mistakes

Learners may overlook the bedside monitor i.e., forgetting to verbalize or connect the cardiorespiratory (CR) monitors and cycle blood pressure measurements on regular intervals

1. Learners can forget to obtain bedside point of care glucose: Important to reinforce POC glucose with any tired or ill appearing pediatric patient
2. Learners may be unable to recognize the worsening clinical status after Intravenous Fluid bolus: Reinforce re-evaluation after every intervention, scale back on the “standard” 20 cc/kg bolus for cardiac etiologies
3. Learner does not recognize that this could be COVID-19. They may not wear appropriate PPE and do not recognize that this could be the inflammatory syndrome associated with COVID-19. Have found that in certain cases the nurse needs to tell the learners that this seems like what she has heard on the TV about that rare syndrome in kids with COVID-19.
4. Learners may use inappropriate or inadequate Rapid Sequence Induction (RSI) medications: Determine the rationale behind the chosen RSI medications for a hypotensive patient in cardiogenic shock, preferably choose ketamine as the induction agent
5. Learners may overlook the changes on cardiorespiratory monitor which lead to diagnosis of VT. Once established the VT diagnosis, check for pulse and manage it based on PALS algorithm
6. Learners may not finalize the disposition of the patient: PICU or ECMO center
